# A neural field model of decision making in the posterior parietal cortex

**DOI:** 10.1186/1471-2202-12-S1-P116

**Published:** 2011-07-18

**Authors:** Christian Klaes, Sebastian Schneegans, Gregor Schöner, Alexander Gail

**Affiliations:** 1 Sensorimotor group, German Primate Center – Leibniz Institute for Primate Research, Göttingen, 37077, Germany; 2 Bernstein Center for Computational Neuroscience, Göttingen, Germany; 3 Institute for Neural Computation, Ruhr University Bochum, Bochum, 44780, Germany

## 

The process of decision making often involves incomplete information about the outcome of the decision. In order to plan goal-directed reaching, it is necessary to combine sensory information about goal positions with information about the current behavioral context to select an appropriate action. A central role in this process is attributed to the posterior parietal cortex (PPC), which has been associated with value based selection of action and perceptual decision making. As an underlying mechanism, it has been proposed that the selection and specification of possible actions are not two distinct, sequential operations, but that instead the decision for an action emerges from the competition between different movement plans [1].

**Figure 1:**
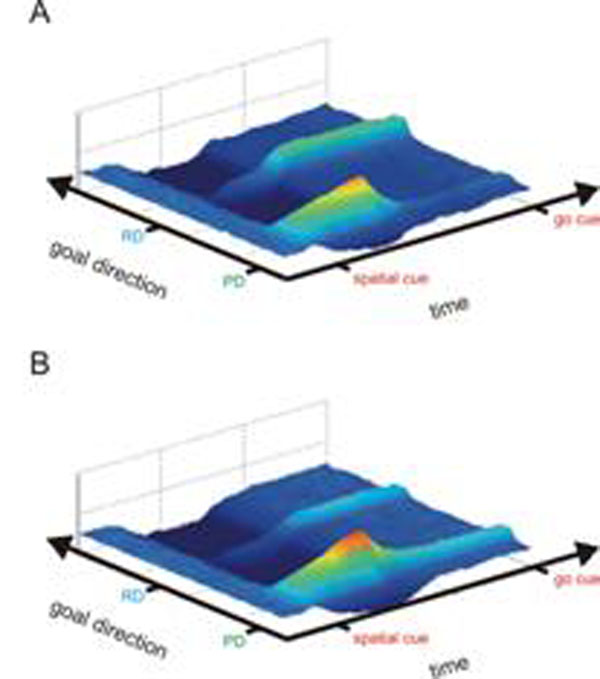
Activity in the PPC layer of the model. A spatial cue is presented at the probe direction (PD). The remapped direction (RD) is the position that the model previously learned to be the valid goal, when an additional contextual cue was presented. The time axis shows the memory period from onset of the transient spatial cue until just before the goal is to be selected (go cue). When exposed to 80 % remapped motor goals and 20 % direct mapped goals, the model develops a bias towards the remapped direction (RD), when tested in a free choice trial (A). If the ratio is 50/50, both potential motor goals are equally represented (B).

Here, we present a neural field model [2] to describe the dynamics of action selection in the PPC, developed in parallel with an electrophysiological study in monkeys [3]. The task required rule-based spatial remapping of a motor goal, which was indicated by a spatial cue, depending on a contextual cue. The model can learn the context-dependent remapping task via an implemented Hebbian-style learning rule. It is trained from a pre-structured initial state (with default cue-response mapping behavior), using a training procedure that emulates the training procedure of the monkeys. The trained model developed activity patterns and neuronal tunings consistent with the empirical data. We then examined how actions are planned in the absence of an explicit rule, i.e. with no contextual cue. In this case the model showed a decision bias towards one goal (Fig. 1A) or an equal representation of both potential goals (Fig. 1B), depending on the input statistics during training. The model remained susceptible to later experience and changes of the reward schedule.

This matches the observations in monkeys performing the same task and it provides an account for the formation of action plans under ambiguous conditions. The field model provides an integrated account for the operations of sensorimotor transformations, working memory, and action selection required for decision making.
